# Chronic vagus nerve stimulation improves left ventricular function in a canine model of chronic mitral regurgitation

**DOI:** 10.1186/s12967-014-0302-2

**Published:** 2014-11-04

**Authors:** Haiwen Yu, Min Tang, Jun Yu, Xiaohong Zhou, Lepeng Zeng, Shu Zhang

**Affiliations:** State Key Laboratory of Cardiovascular Disease, Fuwai Hospital, Peking Union Medical College and Chinese Academy of Medical Sciences, Beijing, 100037 People’s Republic of China; Cardiac Rhythm Disease Management, Chinese Branch of Medtronic Inc., Shanghai, China

**Keywords:** Autonomic nervous system, Vagus nerve stimulation, Mitral valve insufficiency, Heart failure

## Abstract

**Background:**

Autonomic dysfunction, characterized by sympathetic activation and vagal withdrawal, contributes to the progression of heart failure (HF). We hypothesized that chronic vagus nerve stimulation (VNS) could prevent left ventricular (LV) remodeling and dysfunction in a canine HF model induced by chronic mitral regurgitation (MR).

**Methods and results:**

After the MR inducing procedure, 12 survived canines were randomly divided into the control (n = 6) and the VNS (n = 6) groups. At month 2, a VNS stimulator system was implanted in all canines. From month 3 to month 6, VNS therapy was applied in the VNS group but not in the control group. At month 6, compared with the control group, the canines in VNS group had significantly higher cardiac output (2.3 ± 0.3 versus 2.9 ± 0.4 L/min, P < 0.05, LV forward stroke volume (20.1 ± 3.7 versus 24.8 ± 3.9 ml, P < 0.05), and end-systolic stiffness constant (2.2 ± 0.3 versus 2.7 ± 0.3, P < 0.05). NT-proBNP and C-reactive protein were decreased significantly in the VNS group. However, no statistical difference was found in LV ejection fraction, LV end-diastolic dimension, LV end-diastolic volume, myocyte cross-sectional area, or collagen volume fraction between two groups.

**Conclusions:**

Chronic VNS therapy may ameliorate MR-induced LV contractile dysfunction and improve the expression of biomarkers, but has less effect in improving LV chamber remodeling.

## Background

One of the key features of chronic heart failure (CHF) is the autonomic sympathetic/parasympathetic imbalance, which is usually characterized by excessive sympathetic drive accompanied by parasympathetic withdrawal [1,2]. It directly affects the heart and blood vessels, and indirectly modulates the blood volume through the renin-angiotensin-aldosterone (RAA) system and vasopressin signaling. Extensive clinical evidences have demonstrated that suppressing sympathetic activity using pharmacological agents such as beta-adrenergic receptor blockers could reverse sympathetic/parasympathetic imbalance, and thus reduce mortality and morbidity in patients with CHF [3,4]. On the other hand, reversing the sympathetic/parasympathetic imbalance by enhancing parasympathetic activity through vagal nerve stimulation (VNS) becomes an obvious potential therapeutic approach. The effect of chronic VNS on cardiac remodeling and long-term survival in various animal model of chronic HF has been evaluated. In a rat myocardial infarction model [5], chronic VNS attenuated HF progression, prolonged survival time and improved the expression of biomarkers such as C-reactive protein (CRP). In both high-rate pacing and post-infarction HF canine models, chronic VNS attenuated cardiac remodeling and maintained cardiac function [6,7]. A non-randomized chronic VNS clinical trial on 32 NYHA class II–IV heart failure patients has demonstrated that chronic VNS in CHF patients with severe systolic dysfunction is safe and tolerable, and may improve patients’ quality of life and left ventricular (LV) function [7].

Cardiac sympathetic activation is increased in patients with CHF due to mechanical overload. By suppressing cardiac sympathetic activities, β-blockade had been demonstrated to be an effective treatment for contractile dysfunction caused by valvular heart disease [8]. However, whether enhancing cardiac parasympathetic activities by VNS can be an alternative effective treatment for such contractile dysfunction is unknown. This study was designed to test the hypothesis that chronic VNS can attenuate LV remodeling and improve LV function for CHF caused by chronic mitral regurgitation (MR).

## Methods

### Study design

The study was performed in accordance with the Guide for the Care and Use of Laboratory Animals published by the US National Institutes of Health (NIH Publication, revised 1996) and was approved by the animal research committee of Fuwai hospital, Peking Union Medical College.

Mitral valve regurgitation was induced by chordae rupture using a fluoroscopic guided catheterization method in 16 conditioned, 17 to 24 kg, male/female mongrel canines. Twelve canines survived at week 1 after the procedure and were randomized into the control (n = 6) and VNS (n = 6) groups. At the month 2, a bipolar cuff electrode was implanted around the right cervical vagus nerve and connected to a customized implantable pulse generator (Zhang De Scientific Corporation, Shanghai, China) in all canines. All animals were then closely monitored for a month without VNS therapy to allow sufficient recovery from the implantation procedure. At the month 3, consistent contractile dysfunction was observed in all 12 animals, which is consistent with previous reports [9]. The VNS therapy were turned on in the animals of VNS group from the month 3 to month 6, while the VNS therapy were kept off in the animals of the control group during the same period. Ventriculography, Doppler echocardiography, electrocardiograph and hemodynamic data were collected at baseline, month 3 before VNS therapy, and month 6 respectively. During data collection, VNS was temporarily turned off 10 minutes right before the collection and remained off during the procedure of cardiac catheterizations and hemodynamic evaluations. Blood samples were also collected at the same time point. At the month 6, the animals were humanely euthanized under deep anesthesia and the related cardiac tissues were dissected and stored in 10% formalin for histological examination.

### Creation of mitral regurgitation

Mitral regurgitation was created using the close-chest chordae rupture technique as described previously [8,10]. Briefly, after baseline measurements, a customized Swarz sheath was inserted through the carotid artery into the LV guided by fluoroscopic imaging. A biopsy clamp was used to rupture chordae tendineae through the sheath. The wedge pressure, aortic pressure, pulse rate, and forward stroke volume were assessed after each chordae rupture. When pulmonary capillary wedge pressure (PCWP) rose to 20 mm Hg and forward stroke volume decreased to 50% of the baseline , a ventriculogram was taken to confirm the creation of severe MR and the regurgitant fraction was calculated to quantify the amount of MR.

### Implantation of devices and vagus nerve stimulation

Right cervical vagus nerve stimulators were implanted in both groups as previously described [7]. Briefly, a cervical VNS cuff electrode was placed around the right cervical vagus nerve. The electrode was connected to a nerve stimulator buried in a pocket at the neck area. In the VNS group, the bi-directional VNS (20 Hz, pulse width 0.5 ms, both efferent and afferent) was turned on at the month 3 and delivered continuously from the month 3 to the month 6 until the end of the study. A telemetric device (Zhang De Scientific Corporation, Shanghai, China) was also implanted for remote ECG monitor when right cervical vagus nerve stimulators were implanted. The VNS intensity (1.0 to 3.0 mA, average 2.1 ± 0.5 mA, duty cycle 10s and 30s off) was individually titrated twice a week to allow 15 beats/min spontaneous sinus rate reduction.

### Catheterization

Canines were anesthetized with intravenous injection of pentobarbital (25 mg/kg). Then, animals were mechanically ventilated with oxygen (2 L/min) and anesthesia was maintained with halothane after intubation. Right or left femoral artery was used for catheterization. Two pigtail catheters were introduced into the LV and the ascending aorta. Mercury calibrated water-filled transducers were connected to the pigtail catheters. A multilumen thermodilution catheter was inserted into the left jugular vein and positioned in the pulmonary artery to measure cardiac output, pulmonary artery and pulmonary capillary wedge pressures. A 25-mm balloon catheter was introduced into the inferior vena cava through the same vein to monitor LV volume and pressure by inflating and deflating the balloon during ventriculography. After baseline measurements, a standard left ventriculogram using nonionic contrast (iohexol) was performed. After a 15-min equilibration, a second ventriculogram was taken and synchronized with balloon deflation so that beat-by-beat increases in both LV pressure and volume were recorded simultaneously [11]. The pressure-volume data produced from these alterations were used to construct the index of LV contractile function.

### Assessment of in vivo contractile function

Contractility is the inherent capacity of the myocardium to contract independently of changes in the preload or afterload. In vivo contractile function was assessed using the end systolic stiffness constant (K index). The K index has been investigated extensively in the past and its limitations are relatively well known. It has correlated well with an independent standard of contractile function-sarcomere function in isolated cardiomyocytes [9,12]. Data simultaneously collected from angiograms and high-fidelity manometers were used to develop such relationship.

LV volume and mass were determined by the area-length method which was previously demonstrated to be able to provide accurate LV volume and mass in dogs with MR [13]. The regurgitant fraction (RF) was calculated as: RF (in %) = [(SV_a_-SV_f_)/SV_a_] × 100, where SV_a_ is stroke volume measured by angiography and SV_f_ is forward stroke volume derived from actual cardiac output measured by thermodilution and heart rate. Wall stress was calculated using Mirsky’s formula [14]. The end-systolic stiffness constant (K-index) was determined by fitting end-systolic wall stress and end-systolic wall thickness using the curvilinear equation as: ơ = *Ce*^κln(1/H)^, where ơ is end-systolic wall stress, C is a constant, κ is the end-systolic stiffness constant and ln(1/H) is the natural logarithm of the reciprocal of wall thickness [12].

### Echocardiography

Transthoracic 2-dimensional echocardiography was performed when canines were consciously lying on the table in the left lateral decubitus position. Standard 2-dimensional short- and long-parasternal views, as well as 4-chamber, 2-chamber, and 3-chamber apical views were obtained via standard procedure using a 3S transducer coupled to a Vivid 7 echocardiographic machine (GE Medical, Milwaukee, Wis). LV end-diastolic and end-systolic volume, and ejection fraction were measured three times using a Simpson’s biplane method. The averages were used in the final data processing.

### NT-proBNP and CRP levels in plasma

Venous blood samples were collected and placed in ice-chilled tubes coated with EDTA. Plasma was separated for 15 minutes by 3000 rpm centrifugation at 4°C and stored at −80°C. The levels of NT-proBNP and CRP were measured by enzyme-linked immunosorbent assay (ELISA, ALPCO, Salem, NH, USA).

### Histomorphometric assessment

#### Myocyte cross-sectional area

Paraffin-embedded tissue samples were cut at 5 μm thickness and stained with hematoxylin and eosin (H&E). The cardiomyocytes of which cross-sections contain a centrally located nucleus with intact cellular membrane [15] from H&E sections was selected for the size assessment using NIH Image J software (NIH Image, Bethesda, MD). The mean cross-sectional area (CSA) from approximately 200 cardiomyocytes from each animal was calculated.

#### Collagen analysis

Epicardial and endocardial sections from the mid-LV wall were obtained and paraffin-embedded after the fixation, 5-μm thick paraffin sections were stained with Sirius red F3BA. Interstitial collagen was identified using light microscopy at high power (×40 objective, ×1,600 total magnification). To evaluate the collagen volume fraction(CVF), about 3 sections from each animal were picked and 30–40 randomly selected fields in each section were used to calculate the mean ratio of connective tissue area to the total tissue area [16]. The double blindfold measurements were taken.

### Statistical analysis

Data are expressed as mean ± SEM. Both two-sided One-way ANOVA and two-sided Holm-Sidak post hoc *t*-test were performed using SPSS 16.0 statistical software). Proper and necessary data transformations (logarithmic or square root) were applied for non-normal distributed data prior to the statistical analysis. *P value less than* 0.05 was considered to be statistically significant in the test.

## Results

### Hemodynamic and remodeling

All canines were survived through the 6 months experimental period. The related LV function and hemodynamic data were summarized in Table 1. Briefly, there was no significant difference for the MR measurement at the baseline and month 3 between the control group and VNS group. At month 6, the heart rate (HR) was significantly decreased in VNS group compared with controls, (Control 139 ± 11 beats/min vs. VNS 122 ± 7 beats/min, P < 0.05), LV mass/body weight (BW) (Control 5.8 ± 0.3 g/kg vs. VNS 6.4 ± 0.3 g/kg), and PCWP (Control 17.1 ± 1.9 g/kg vs. VNS 11.2 ± 1.7 g/kg). The control group had less but not statistically significant LV end-diastolic dimension (LVEDD), dP/dt_max_, LV end-diastolic volume (LVEDV) than those of the VNS group. There was no significant difference systemic blood pressure and RF between two groups at all observation periods.

**Table 1:** **Left ventricular function and hemodynamics in MR: effect of VNS**

**Measurement**	**Arm**	**Baseline**	**3 month**	**6 month**
HR (beats/min)	Control	103 ± 6	135 ± 9	139 ± 11
	VNS	106 ± 6	137 ± 10	122 ± 7*
mAoP (mm Hg)	Control	99 ± 5	85 ± 8	80 ± 8
	VNS	97 ± 5	86 ± 9	78 ± 9
PCWP (mm Hg)	Control	5.9 ± 0.4	17.4 ± 1.4	19.1 ± 1.9
	VNS	5.7 ± 0.5	18.6 ± 1.6	13.2 ± 1.7*
dp/dt_max_ (mm Hg/s)	Control	2543 ± 226	1925 ± 255	1782 ± 278
	VNS	2496 ± 209	1911 ± 243	1938 ± 295
RF (%)	Control	58 ± 3	63 ± 4	61 ± 5
	VNS	59 ± 3	65 ± 4	61 ± 6
LVEDD (cm)	Control	3.5 ± 0.2	4.9 ± 0.3	5.8 ± 0.3
	VNS	3.4 ± 0.2	4.8 ± 0.3	6.1 ± 0.4
LVESD (cm)	Control	1.9 ± 0.1	2.6 ± 0.1	3.1 ± 0.3
	VNS	1.8 ± 0.1	2.5 ± 0.1	3.2 ± 0.3
LVEDV (ml)	Control	43 ± 4	84 ± 10	110 ± 12
	VNS	42 ± 4	85 ± 10	117 ± 13
LVESV (ml)	Control	19 ± 3	30 ± 8	41 ± 10
	VNS	18 ± 3	28 ± 8	42 ± 11
LV mass/BW (g/kg)	Control	3.8 ± 0.2	5.5 ± 0.3	5.8 ± 0.3
	VNS	3.7 ± 0.2	5.4 ± 0.3	6.3 ± 0.3*

Values presented as mean ± SEM. *P < 0.05 compared with the control.

HR = heart rate; mAoP = mean aortic pressure; PWCP = Pulmonary capillary wedge pressure; RF = regurgitant fraction; LV = left ventricle/ventricular; EDD = end-diastolic dimension; EDV = end- diastolic volume; ESD = end-systolic dimension; ESV = end-systolic volume; BW  = body weight.

**Figure 1:**
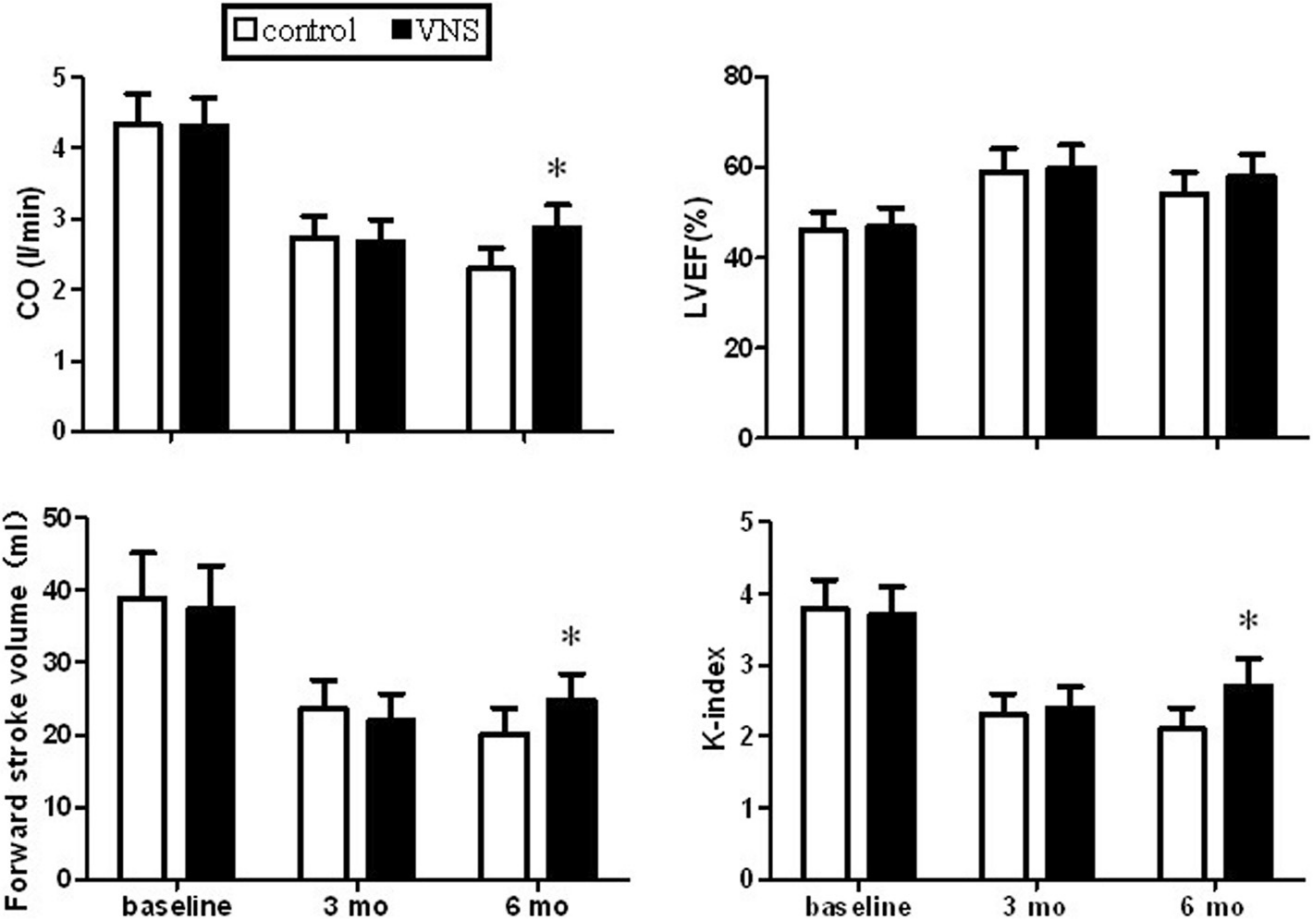
**Left ventricular ejection fraction, K-index, cardiac output and forward stroke volume at baseline, at 3- and at 6-mo of MR in control and VNS treated dogs.** *P < 0.05 versus control group.

The summary of LV ejection fraction (LVEF), K-index, cardiac output (CO) and forward stroke volume was showed in Figure 1. There was a significant difference (P < 0.05) in the K-index, CO and forward stroke volume at 6 month between the control group and the VNS group. The LVEF in control group was lower than that in the VNS group but just missed the statistically significant.

### NT-proBNP and CRP levels in plasma

The plasma NT-proBNP levels increased significantly in both the control group and the VNS group compared to the baseline while no significant changes between both groups at month 3 after the MR. However, at month 6, the NT-porBNP levels were significantly lower in the VNS group than the levels in the control group.

**Figure 2:**
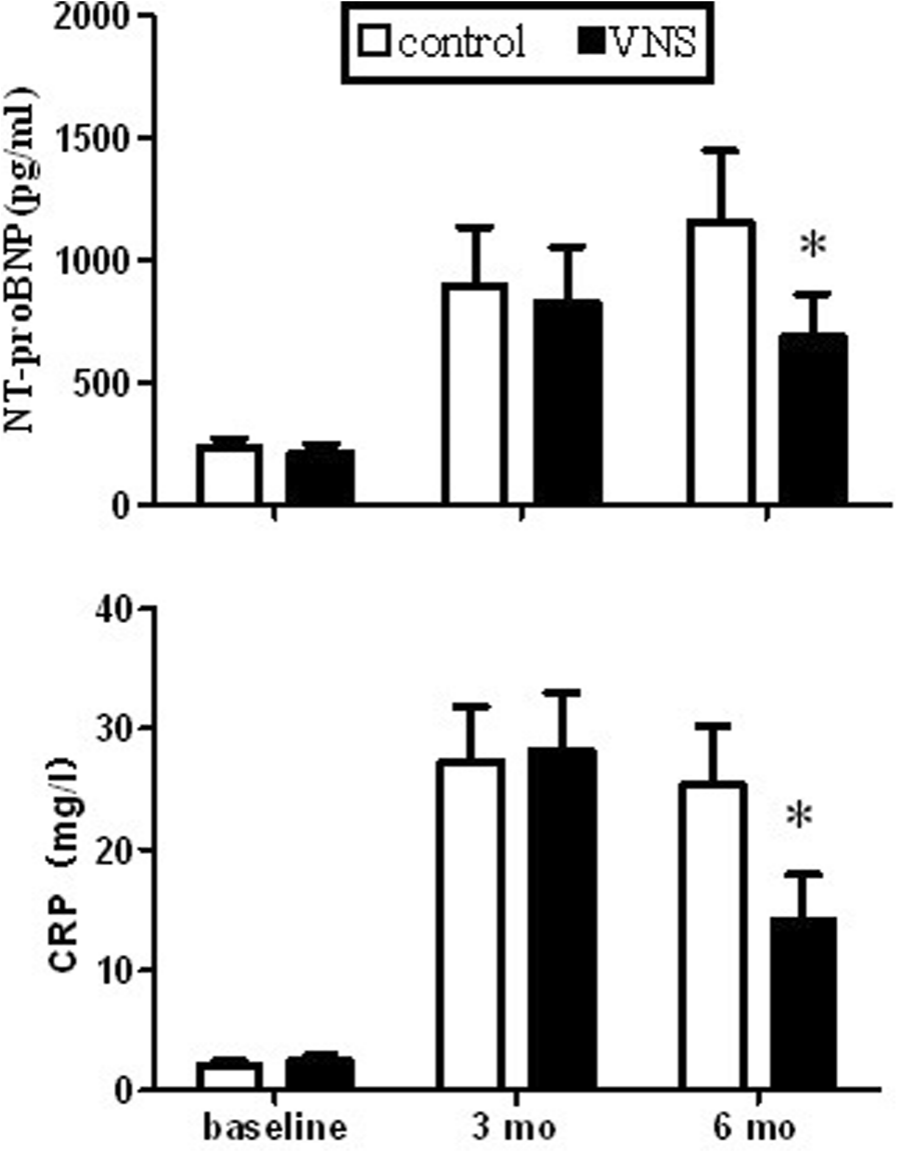
**Plasma C-reactive protein and NT-proBNP levels at baseline, at 3- and at 6-mo of MR in control and VNS treated dogs.** *P < 0.05 versus control group.

Plasma CRP level also increased at 3 months of MR compared to the baseline while no significant difference between both groups. However, the CRP level was lower in the VNS group compared with the the control group at 6 months significantly (Figure 2).

### Histomorphometric assessment

**Figure 3:**
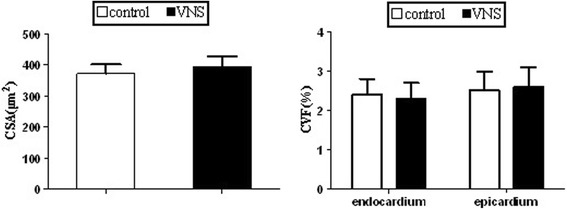
**Cross-sectional area at 6-mo of MR and interstitial collagen volume in both the endocardium and epicardium at 6-mo of MR in control and VNS-treated dogs.** *P < 0.05 versus control group.

As shown in Figure 3, there was no significant difference in LV cardiomyocyte CSA, endocardial and interstitial epicardial CVF between the control group and the VNS group at the month 6.

## Discussion

The present study investigated the effects of chronic VNS on HF development in a chronic MR induced HF canine model. There were two major findings of this study. Firstly, VNS preserved LV contractile function and favorably altered cardiac biomarkers. To our best knowledge this is the first time to report that VNS is effective in the treatment of contractile dysfunction result from experimental chronic MR. Secondly, VNS in the present study failed to prevent the LV from hypertrophic and eccentric remodeling.

### Therapeutic effects in LV contractile function

The results from several pre-clinical investigations [5-7] supported the concept of VNS as a therapeutic approach to treatment CHF. However the effect of VNS on chronic MR model remains unknown. It was unclear whether VNS can favorably modify the underlying pathophysiology of chronic MR. Our study showed that three month VNS substantially improved forward stroke volume, CO, dP/dt_max_ and K-index compared to non-treated control group after three month chronic MR. Furthermore, the PCWP and plasma NT-proBNP level were were remarkably decreased in the VNS group at month 6 compared with controls.

Conflicting results on the usage of renin angiotensin system (RAS) blockade and β-blockade therapy in MR model have been reported previously [11,17]. The inconsistence is partially explained by the reason that reload or afterload reduction may actually increase the prolapse and severity of MR [18] in the chronic MR heart failure model. Since VNS dose not affect systemic blood pressure level, it might be a better therapy to MR by avoiding such adverse condition.

Some of previous pre-clinical work [19] suggested that the timely delivery of VNS aligning with physiological cardiac cycles might be a critical factor for the success of VNS. For example, a VNS was delivered at a fixed interval within the cardiac cycle sensed by implanted cardiac lead. A more recent study suggested that VNS can provide a therapeutic benefit without delivering the stimulus at a fixed interval within the cardiac cycle [6]. In our study, we did not implant cardiac lead and deliver stimulus at a fixed interval within the cardiac cycle, these results provided further evidence that the VNS timing within the cardiac cycle is not necessary to achieve the therapeutic effects.

### Possible mechanisms underlying the effects in HF

One of the limitations of the present investigation is that it did not provide clear mechanisms on how VNS preserved LV systolic function during CHF with MR. In general, CHF is characterized by a deregulation of the immune response, and autonomic imbalance accompanied by concomitant abnormalities of reflex cardiorespiratory control. VNS can reverse autonomic sympathetic/parasympathetic imbalance and improve ventricular function and pumping efficiency by slowing HR. A previous study found that β-blockers ameliorate LV contractile dysfunction in experimental chronic MR by restoring cellular contractile elements [8]. However, this improvement did not occur when bradycardia was prevented by atrial pacing [20], suggesting that the LV dysfunction that develops in experimental chronic MR is HR dependent.

Previous studies also suggested that VNS can suppress systemic inflammatory response through “the cholinergic anti-inflammatory pathway” [21,22], which may be beneficial in HF treatment. In the present study, the plasma CRP level was lower in the VNS group at the month 6 than that in the control group, supporting the anti-inflammation theory.

### Therapeutic effects in LV remodeling

Currently, there is no single or multiple drug therapies available for the treatment of chronic MR that prevents adverse LV remodeling [23]. In the present study, we failed to observe VNS improve LV remodeling of chronic MR at month 6.

The canine MR model is characterized by absence of fibrosis and dissolution of the fine collagen weave [16]. The improvement in epicardial matrix components does not prevent further eccentric remodeling of the LV, suggesting that the extracellular matrix(ECM) of the entire wall is important in maintaining LV geometry [24]. Loss of the fine collagen weave destroys the structural support of the ECM that is essential for maintaining the normal LV chamber geometry (30). In the present study, VNS did not improve epicardial and endocardial collagen in the VNS group compared to the untreated control group. Thus, the failure to observe a favorable effect of VNS for LV reverse remodeling may be explained partially by that the VNS failed to prevent the loss of endocardial collagen.

A limitation to translating the present investigations to the clinical setting is creating MR by chordae rupture in our experimental model. This model may not similar to more gradually produced MR which might occur in rheumatic heart disease.

## Conclusions

Chronic VNS therapy ameliorates MR-induced LV contractile dysfunction and improves biomarkers of NT-proBNP and CRP. These findings provided additional supportive evidence for VNS therapy as promising therapy for CHF. In addition, the VNS might preserve LV systolic function by slowing HR during CHF.
